# Prepregnancy Physical Activity in relation to Offspring Birth Weight: A Prospective Population-Based Study in Norway—The HUNT Study

**DOI:** 10.1155/2013/780180

**Published:** 2013-01-29

**Authors:** Silje Krogsgaard, Sigridur L. Gudmundsdottir, Tom I. L. Nilsen

**Affiliations:** Department of Human Movement Science, Norwegian University of Science and Technology, 7491 Trondheim, Norway

## Abstract

*Background*. The objective was to examine the association between prepregnancy physical exercise and offspring birth weight and to assess the combined association of pre-pregnancy body mass index (BMI) and physical exercise on birth weight. *Methods*. The study included 2,026 women aged 20–39 years participating in the Norwegian HUNT study and linked with the Medical Birth Registry. We calculated mean differences in birth weight and odds ratios (ORs) for a macrosomic infant (i.e., birth weight >4000 g) using linear and logistic regression analysis. *Results*. There was no clear association between leisure time physical exercise and mean birth weight. Women who reported no exercise had reduced risk of a macrosomic infant (OR, 0.6; 95% confidence interval (CI), 0.4–0.9) compared to women with a high exercise level. Overweight (BMI ≥ 25.0 kg/m^2^) was associated with an OR of 1.9 (95% CI, 1.2–2.9) for a macrosomic infant among women who reported low exercise levels, whereas the OR was 1.2 (95% CI, 0.8–1.8) among women with higher exercise levels. *Conclusion*. There was some evidence that women who reported no exercise before pregnancy had lower risk for a macrosomic infant than women who exercised. Pre-pregnancy BMI was positively associated with birth weight and risk of macrosomia but only among the least active women.

## 1. Introduction

The proportion of women giving birth to large infants has increased around the world [1, 2], most likely because of the rising rates of maternal overweight and obesity [3–7]. Whereas consequences of low birth weight may include infant mortality and morbidity [8], high birth weight has been related to increased risk for caesarean section, chorioamnionitis, fourth degree perinatal lacerations, postpartum haemorrhage, shoulder dystocia [9–11], and low Apgar score [12]. Additionally, high birth weight has been positively associated with obesity [13] and type 2 diabetes [14] in adulthood.

Previous studies have reported that physical activity in pregnancy is related to foetal growth rate and birth weight [15, 16], and that physically active women have a reduced risk of delivering a large infant [17, 18], possibly by increased insulin sensitivity [6]. However, not all studies have reported consistent inverse associations between physical activity in pregnancy and birth weight [19–22]. Although women who exercise regularly before pregnancy are more likely to continue to exercise during pregnancy [23–25], few studies have examined the associations between prepregnancy physical activity and birthweight, and the results have been inconsistent [18, 20, 26, 27]. 

In this prospective study in Norway, we have utilized data on women participating in a population-based health study linked with information from the Medical Birth Registry to study the association between maternal pre-pregnancy physical exercise and offspring birth weight. Additionally, we explored the combined association of pre-pregnancy body mass index and physical exercise on birth weight.

## 2. Materials and Methods 

### 2.1. Study Population

The Nord-Trøndelag health study (The HUNT study) is a large population-based health study conducted in the county of Nord-Trøndelag, Norway. The HUNT study is a collaboration between HUNT Research Centre (Faculty of Medicine, Norwegian University of Science and Technology NTNU), Nord-Trøndelag County Council, Central Norway Health Authority, and the Norwegian Institute of Public Health. It constitutes three consecutive cross-sectional waves; the first was conducted in 1984–1986 (HUNT 1), the second in 1995–1997 (HUNT 2), and the third in 2006–2008 (HUNT 3). For the purpose of the present study, we have used information from the first wave (HUNT 1). In HUNT 1, 87,285 persons aged ≥ 20 years were invited to participate, and 77,216 (88.5%) accepted the invitation, filled in questionnaires, and attended a clinical examination (37,826 men and 39,390 women). A more detailed description of participation, method, and procedures of the HUNT study can be found elsewhere [28]. 

 For the purpose of the current study, we first selected all 3,739 women aged 20–39 years at participation in HUNT 1 (1984–1986) who gave birth to at least one child during a five year period after participation. Of these 3,739 women, we excluded 232 women due to pre- (<37 weeks) or postterm (>44 weeks) delivery, 32 women with multiple births, 10 women with gestational diabetes, 118 women with preeclampsia, 831 women without information of physical activity, body mass index, or gestational age, and 490 women who gave birth within 10 months after participation (i.e., possibly pregnant at the time of participation). This left 2,026 women available for statistical analysis.

### 2.2. Study Variables

Information on the offspring was obtained by a linkage to the Medical Birth Registry of Norway. These data were obtained for the first child born during five years after participation in HUNT. The main outcome variable was newborn birth weight, measured in grams (g), first analyzed as a continuous variable and then as a dichotomized variable using 4,000 g as cutoff. Macrosomia was defined as birth weight at or above 4,000 g [29]. 

 Leisure time physical exercise was assessed using three questions. In the first question, the participants were asked to report how many exercise sessions (e.g., walking, skiing, swimming, or other sports) they usually had during a week, with five response options (0, <1, 1, 2-3, and ≥4 times; coded 1–5). If the participants reported exercising at least once a week, they were also asked to report the average duration (<15, 15–30, 31–60, and >60 minutes; coded 1–4) and intensity (light, moderate, and to exhaustion; coded 1–3) of the activity. Among participants who reported exercising at least once a week, a summary score of frequency, duration, and intensity was calculated according to the following equation: 1/5  ∗  frequency + 1/4  ∗  duration + 1/3  ∗  intensity. This procedure intended to give equal weight to each component of physical activity and resulted in a maximum score of 1.0 for each of the three components of the summary score. The median score value of 1.97 (range, 1.2–3.0) was then used as a cut-off to classify women into two categories of score values (±median). This information was used to construct a variable of total physical exercise with four unique categories: (1) no activity, (2) low activity (<1 session per week), (3) medium activity (<median score value), and (4) high activity (≥median score value) [30].

 Body mass index (BMI) was computed from standardized measures as weight divided by the squared value of height (kg/m^2^) and categorized into two groups: BMI < 25.0 kg/m^2^ and BMI ≥ 25.0 kg/m^2^ (i.e., overweight or obese) [31]. 

### 2.3. Statistical Analysis

We used linear regression to analyze the association between measures of leisure time physical exercise and mean birth weight. We also calculated odds ratios (OR) for having a macrosomic infant (≥4,000 g) in different categories of leisure time physical exercise using logistic regression. Precision of the estimated associations was assessed by a 95% confidence interval (CI). Women who reported the highest activity level were used as the reference category in all analysis. The following variables were considered as potential confounders in the analysis; age (20–24, 25–29, 30–34, and 35–39 years), smoking (never, former, and current), frequency of alcohol consumption during the past 2 weeks (none, 1–4 times, ≥5 times, abstainer, and unknown), education (<10, 10–12, >12 years, and unknown), marital status (unmarried, married, and previously married), and parity (primiparous, 1-2 children, and 3–6 children). Covariates were removed from the model if there was no meaningful difference between adjusted and unadjusted estimates. All estimates were adjusted for maternal age, smoking, and parity. Tests for trend across categories of leisure time physical exercise were conducted by treating the categories as an ordinal variable in the regression model. 

Since maternal BMI could be both an effect modifier and on the causal pathway between exercise and birth weight, BMI was not included as a confounder in the primary analyses. However, additional analysis was conducted for the combined associations of prepregnancy BMI and total leisure time physical exercise in relation to birth weight, using linear and logistic regression as described earlier. We also included a product term of BMI (<25 versus ≥25 kg/m^2^) and exercise level (no or low activity versus medium or high activity) to assess possible interaction between the two variables and as well as stratified the analyses of physical exercise on the two BMI groups.

All statistical analyses were performed using the statistical software SPSS for Windows, version 17.0.

The study was approved by the Regional Committee for Ethics in Medical Research. All eligible participants received a written invitation with information about the study, and all participants gave their consent by filling in and returning the first questionnaire that was mailed together with the invitation. 

## 3. Results

Descriptive characteristics of the study population are presented in Table 1. Mean baseline maternal age among the 2,026 women in the study was 26.9 years, whereas mean birth weight of their offspring was 3,620 g (SD, 502), and a total of 416 (20.5%) newborns weighed 4,000 g or more (i.e., had macrosomia). 

Table 2 presents results from linear regression showing that there was no clear association between maternal leisure time physical exercise and mean birth weight of their offspring, neither in relation to number of exercise sessions per week (*P* trend, 0.49) nor in relation to total amount of exercise (*P* trend, 0.56). Correspondingly, results from logistic regression presented in Table 3 provide no consistent evidence for an association between maternal physical exercise and risk of a macrosomic infant. However, women who reported being inactive before pregnancy had a lower risk of giving birth to an infant with excessive birth weight (OR, 0.6; 95% CI, 0.4–0.9) compared to women with a high total exercise level (Table 3). 

The combined association of pre-pregnancy BMI and leisure time physical exercise in relation to birth weight is shown in Table 4. Women who were overweight (BMI ≥ 25.0 kg/m^2^) before pregnancy and reported no or low leisure time physical exercise gave birth to infants with significantly higher mean birth weight (134 g; 95% CI, 41.0–227.3) and had a higher risk for a macrosomic infant (OR, 1.9; 95% CI, 1.2–2.9), compared to women with BMI < 25.0 kg/m^2^ who had a medium or high exercise level. In additional analysis stratified by body mass index, overweight women (BMI ≥ 25.0 kg/m^2^) who reported no or low exercise levels had significantly higher offspring birth weight (132 g; 95% CI, 20.4–243.7) and higher odds ratio for a macrosomic infant (OR, 2.0; 95% CI, 1.1–3.5) than women who reported a medium or high exercise level (data not shown). However, there was no statistically significant interaction between BMI and total exercise level (*P* = 0.08). 

## 4. Discussion 

In this large prospective study of Norwegian women, we found no clear association between reported leisure time physical exercise level before pregnancy and offspring birth weight. There was some evidence that inactive women had a slightly lower likelihood of giving birth to a child with excessive birth weight than more physically active women, but the small numbers of inactive women call for a cautious interpretation. Analysis of the combined association of BMI and exercise on birth weight showed that women with a BMI ≥ 25.0 kg/m^2^ gave birth to infants with significantly higher birth weight than women with a BMI < 25.0 kg/m^2^, but only if they also reported no or low levels of physical exercise. This could suggest that physical exercise to some extent reduces the effect of maternal adiposity on offspring birth weight.

 The suggestive evidence that women who were inactive before pregnancy had lower risk for delivering a macrosomic infant is contradictory to some previous studies. Voldner et al. [20] reported that inactive women (defined as <1 h per week) had almost a threefold higher odds ratio for fetal macrosomia than physically active women (>1 h per week). However, another Norwegian study found no association between frequency of regular exercise before pregnancy and offspring with excessive birth weight (≥90th percentile) [18]. In the present study, those who were classified as inactive reported never engaging in physical exercise, and these women could be more extremely sedentary than inactive women in other studies. Studies have shown that a sedentary lifestyle in pregnancy is associated with lower birth weight [32] and an increased risk of a very low birth weight infant [16]. It has been observed that mothers of very low birth weight infants were less likely to be physically active during their pregnancy [33]. It has been suggested that both excessive and insufficient physical activities in pregnancy are related to an inadequate fetal growth [34], although some women might be advised to be inactive and at rest to reduce the risk of adverse pregnancy outcomes. 

Unlike the present study, some previous studies have shown inverse associations between maternal pre-pregnancy exercise behaviors and offspring birth weight or risk of having excess weight [17, 18], although the results are not entirely consistent [21]. A recent study of leisure time physical activity during pregnancy is more in agreement with the results of the present study. Hegaard et al. [22] found no association with mean birth weight or the risk of giving birth to low (<2,500 g) or high (≥4,500 g) birth weight infants. The inconsistent results in these studies could be due to different measures of physical activity, in addition to the possibility for chance findings in the smaller studies. 

 There is growing evidence that overweight or obesity before pregnancy is a risk factor for macrosomia [3, 12, 35, 36]. The results from the present study suggest that the effects of maternal pre-pregnancy overweight were associated with higher birth weight only among women who reported no or low level of activity. This is contradictory to the findings by Löf et al. [27] who showed that a high pre-pregnancy activity level did not reduce the risk of high birth weight infants in women who were overweight or gained much weight in pregnancy. Nevertheless, physical activity may improve maternal weight control, both before and during pregnancy [37, 38]. 

 The strengths of the present study include the prospective design, the large sample size of women reporting physical activity before pregnancy, and the standardized measures of size at birth obtained from the Medical Birth Registry of Norway. However, some of the categories of physical exercise (e.g., inactive) suffered from small samples size, and this could result in chance findings. Moreover, as in any observational study, residual confounding due to unmeasured (e.g., smoking during pregnancy and gestational weight gain) or unknown factors cannot be ruled out. Since the information on physical activity was self-reported, it could be subject to misclassification, although a validation study has shown acceptable agreement with objective measures [39]. Although pre-pregnancy physical activity has been associated with physical activity level during pregnancy [23–25], it has also been shown that exercise levels decline in pregnancy [24, 25, 40, 41]. Unfortunately, our data did not allow us to examine if pre-pregnancy physical exercise was related to the activity level during pregnancy. 

## 5. Conclusions

There was no clear association between maternal prepregnancy leisure time physical exercise and offspring birth weight. However, the result may indicate that inactive women gave birth to infants with lower birth weight and had lower risk for delivering an infant with macrosomia. The results also show that high maternal pre-pregnancy BMI was associated with higher mean birth weight and increased risk of macrosomia in offspring of physically inactive women, whereas pre-pregnancy BMI was not associated with birth weight and risk of macrosomia among more active women. 

## Figures and Tables

**Table 1:** Characteristics of the study population (*N* = 2,026) according to total leisure time physical exercise.

Characteristic	Total physical exercise level^a^			
	No activity	Low	Medium	High
No participants (% of total)	147 (7.3)	647 (31.9)	641 (31.6)	591 (29.2)
Mean age at baseline, *y *	26.8	27.2	26.7	26.6
Mean body mass index, kg/m²	22.4	22.3	22.5	22.4
Parity (% primiparous)	20.4	28.2	28.5	40.6
Smoking (% current smoking)	53.1	44.0	38.8	31.3
Education (% college/university)	8.1	16.1	20.6	25.4
Alcohol (% not drinking last 2 weeks)	49.7	45.7	46.0	43.1
Marital status (% married)	53.1	55.6	49.5	46.7

^a^Based on a summary score of frequency, duration, and intensity of exercise.

**Table 2:** Maternal pre-pregnancy leisure time physical exercise and mean offspring birth weight from linear regression analyses.

Physical exercise	No. of persons	Mean birth weight (g)	Crude difference	Adjusted^a^ difference (95% CI)	*P* trend^b^
Sessions per week					
None	147	3589.0	−25.2	−26.9 (−137.9 to 84.2)	
<1	647	3632.5	18.3	21.7 (−64.8 to 108.3)	
1	591	3639.7	25.5	31.0 (−56.1 to 118.1)	
2-3	491	3590.4	−23.7	−24.4 (−113.2 to 64.4)	
≥4	150	3614.1	0.0	0.0 (reference)	0.49
Total exercise^c^					
No activity	147	3589.0	−38.9	−53.0 (−141.8 to 35.8)	
Low	647	3632.5	4.6	−3.5 (−58.3 to 51.4)	
Medium	641	3606.9	−21.0	−36.9 (−91.5 to 17.8)	
High	591	3627.9	0.0	0.0 (reference)	0.56

CI: confidence interval.

^a^Adjusted for maternal age (20–24, 25–29, 30–34, and 35–39 years), smoking (never, former, current, and unknown), and parity (primiparous, 1-2 children, and 3–6 children).

^b^ *P* value from trend test when categories were entered as an ordinal variable in the regression model.

^c^Based on a summary score of frequency, duration, and intensity of exercise.

**Table 3:** Odds ratio (OR) from logistic regression for giving birth to a marcosomic infant (i.e., birth weight > 4000 g) in association with maternal pre-pregnancy leisure time physical exercise.

Physical exercise	No. of persons	No. of cases	Crude OR	Adjusted^a^ OR (95% CI)	*P* trend^b^
Sessions per week					
None	147	20	0.7	0.7 (0.4–1.3)	
<1	647	148	1.3	1.3 (0.8–2.1)	
1	591	122	1.1	1.2 (0.7–1.8)	
2-3	491	98	1.2	1.1 (0.7–1.8)	
≥4	150	28	1.0	1.0 (reference)	0.89
Total exercise^c^					
No activity	147	20	0.6	0.6 (0.4–0.9)	
Low	647	148	1.2	1.1 (0.9–1.5)	
Medium	641	128	1.0	0.9 (0.7–1.2)	
High	591	120	1.0	1.0 (reference)	0.65

CI: confidence interval.

^a^Adjusted for maternal age (20–24, 25–29, 30–34, and 35–39 years), smoking (never, former, current, and unknown), and parity (primiparous, 1-2 children, and 3–6 children).

^b^ *P* value from trend test when categories were entered as an ordinal variable in the regression model.

^c^Based on a summary score of frequency, duration, and intensity of exercise.

**Table 4:** Maternal pre-pregnancy body mass index (BMI) and leisure time physical exercise related to mean offspring birth weight from linear regression and odds ratio (OR) from logistic regression for a macrosomic infant (i.e., birth weight > 4000 g).

Combined categories of BMI and exercise^a^	No. of persons	Crude mean difference	Adjusted^b^ mean difference (95% CI)	No. of cases (BW > 4000 g)	Crude OR	Adjusted^b^ OR (95% CI)
<25 kg/m^2^ and medium/high level	1,046	0.0	0.0 (reference)	206	1.0	1.0 (reference)
<25 kg/m^2^ and no/low level	676	−8.6	−8.4 (−55.6 to 38.8)	131	1.0	1.0 (0.8–1.2)
≥25 kg/m^2^ and medium/high level	186	29.8	33.1 (−42.5 to 108.7)	42	1.2	1.2 (0.8–1.8)
≥25 kg/m^2^ and no/low level	118	129.7	134.1 (41.0 to 227.3)	37	1.9	1.9 (1.2–2.9)

CI: confidence interval; BW: birth weight.

^a^Based on a summary score of frequency, duration, and intensity of exercise.

^b^Adjusted for maternal age (20–24, 25–29, 30–34, and 35–39 years), smoking (never, former, current, and unknown), and parity (primiparous, 1-2 children, and 3–6 children).
